# Learning Curve of *Ex Vivo* Liver Resection and Autotransplantation in Treating End-Stage Hepatic Alveolar Echinococcosis: A RA-CUSUM Analysis

**DOI:** 10.3389/fsurg.2021.753968

**Published:** 2021-11-30

**Authors:** Yiwen Qiu, Xianwei Yang, Tao Wang, Shu Shen, Yi Yang, Bin Huang, Wentao Wang

**Affiliations:** ^1^Department of Liver Surgery and Liver Transplantation Center, West China Hospital of Sichuan University, Chengdu, China; ^2^Department of Vascular Surgery, West China Hospital of Sichuan University, Chengdu, China

**Keywords:** risk-adjusted cumulative sum analysis, autotransplantation, liver transplantation, living donor, *ex vivo* resection, alveolar echinococcosis

## Abstract

**Background:** This retrospective study aimed to evaluate the safety and learning curve of *ex vivo* liver resection and autotransplantation (ELRA).

**Methods:** A total of 102 consecutive end-stage HAE patients who underwent ELRA between 2014 and 2020 in West China Hospital were enrolled. The primary endpoint was major postoperative complications (comprehensive complication index, CCI > 26). The ELRA learning curve was evaluated using risk-adjusted cumulative sum (RA-CUSUM) methods. The learning phases were determined based on RA-CUSUM analysis and tested for their association with intra- and post-operative endpoints.

**Results:** The median surgery time was 738 (659–818) min, with a median blood loss of 2,250 (1,600–3,000) ml. The overall incidence of major morbidity was 38.24% (39/102). Risk-adjusted cumulative sum analysis demonstrated a learning curve of 53 ELRAs for major postoperative complications. The learning phase showed a significant association with the hemodynamic unstable time (HR −30.29, 95% CI −43.32, −17.25, *P* < 0.0001), reimplantation time (HR −13.92, 95% CI −23.17, −4.67, *P* = 0.004), total postoperative stay (HR −6.87, 95% CI −11.33, −2.41, *P* = 0.0033), and postoperative major morbidity (HR 0.25, 95% CI 0.09, 0.68, *p* = 0.007) when adjusted for age, disease course, liver function, and remote metastasis.

**Discussion:** *Ex vivo* liver resection and autotransplantation is feasible and safe with a learning curve of 53 cases for major postoperative complications.

## Introduction

*Ex vivo* liver resection and autotransplantation (ELRA) was introduced in recent years to treat end-stage hepatic alveolar echinococcosis (HAE). In contrast to early attempts of treating advanced hepatobiliary malignant tumors in tricky anatomical locations, ELRA has shown promising preliminary results in treating benign or low-grade tumors. End-stage HAE, a zoonotic parasitic disease caused by *Echinococcus multilocularis (Em)* infection (1), is characterized by a single large lesion or multiple lesions that have extensively invaded crucial intrahepatic vessels including retrohepatic vena cava and porta hepatis. Conventional hepatectomy is impossible for treating end-stage HAE because the liver cannot tolerate the length of time required for the reconstruction procedure following resection (2). The abovementioned features made end-stage HAE a rational indication for ELRA.

While ELRA became a promising alternative for treating end-stage HAE, the complexity and safety of this technique hampered its further application. Pioneered by Pilchmayr in 1988 (3), ELRA is a series of procedures involving complete hepatectomy, extracorporeal liver resection followed by hepatic vessel repair, and autologous transplantation of the remnant liver graft.

There is concern that the benefit of ELRA is compromised by frequent surgery-related morbidity caused by insufficient surgical expertise. However, the existing reports on ELRA showed acceptable outcomes regarding perioperative complications (4). Large case series are lacking, and no randomized clinical trials have been conducted. More evidence of feasibility, safety, and especially its learning curve is needed before further introduction of ELRA.

We are the nearest medical center from the AE epidemic area with the highest prevalence rate in the world (5) and one of the earliest centers to explore the use of liver transplantation (LT) (6) and ELRA (7) for the treatment of end-stage HAE, which has helped us acquire considerable experience in the surgical treatment of this disease (8, 9). This single-center series provides the outcomes of the largest cohort of patients who underwent ELRA to date to treat end-stage HAE with the aim of determining the learning curve for this procedure.

## Methods

### Ethics Approval

This study was approved by the Ethics Committee of West China Hospital of Sichuan University (No. 2017-38) and was conducted in accordance with the Declaration of Helsinki. Before surgery, we communicated fully with the patients and their families in their native language and explained the advantages and possible complications of surgery.

### Patients

From January 2014 to December 2020, 318 patients were diagnosed with end-stage HAE in West China Hospital. The diagnostic standard followed the PNM staging system for alveolar echinococcosis (AE) proposed in 2006 by the European Network for Concerted Surveillance of AE and the WHO Informal Working Group on Echinococcosis (IWGE) (10). End-stage HAE was defined as an HAE lesion extending along vessels, including the inferior vena cava (IVC), portal vein (PV), and arteries, with or without neighboring organ invasion and remote metastasis (P4, N1 or N0, M0 or M1). Among them, 102 patients received ELRA according to the following critical features (9): a. involvement of the hepatocaval confluence region, three hepatic veins (HVs), and the IVC or invasion of the secondary or tertiary branches of the PVs and hepatic arteries, all of which required complex reconstruction with a prolonged ischemic time that the liver could not tolerate; and b. good physiological condition, with normal liver and kidney functions and extrahepatic echinococcosis lesions that could be surgically removed or controlled with albendazole. Furthermore, the serum total bilirubin levels were less than twice the upper limit of the normal value, and the estimated remnant liver volume (RLV)-to-standard liver volume (SLV) ratio was at least 0.35.

### Preoperative Assessment

The preoperative assessment procedures have been reported elsewhere (8). In addition to a conventional imaging examination, a three-dimensional (3D) imaging analysis system (CAS; Qingdao Hisense Medical Equipment Co., Ltd., Qingdao, P. R. China) was used to examine the vascular and biliary tract anatomy and the spatial locations of large lesions, conduct virtual resection and calculate the estimated RLV (11). The SLV was calculated following the experience of Urata et al. (12, 13).

The evaluation of lesion anatomy followed our previously devised vascular infiltration classification (9). This classification precisely describes extension of the lesion regarding infiltration into the IVC, HV, and PV, improves comprehension of the anatomy of the lesion and the vasculature and facilitates the planning of reconstruction procedures.

Data on liver function, neighboring organ invasion, remote metastasis, the number and diameter of lesions, the estimated RLV, the SLV, vascular infiltration, and liver function were collected.

### Surgical Technique

The entire series was conducted by the same surgical team led by a senior surgeon with experience with over 300 liver allotransplantations (over 50 living-donor LTs). The technical details of ELRA have been described in our previous report (8). Briefly, the operation was divided into three main parts. First, Procurement of the diseased liver were performed with combined resection of invaded neighboring organs; then, the systemic hemodynamic stability was maintained by a temporarily established IVC and portocaval shunt. In the second part, the liver parenchyma was dissected under hypothermic preservation, and critical conduit structures were carefully preserved for subsequent reconstruction. The final part of the operation included reimplantation of the remnant liver and reconstruction of inflow, outflow, and biliary tract.

### Outcomes

The primary study endpoint was postoperative morbidity and mortality within 90 days, which was recorded in the form of the comprehensive complication index (CCI) (14, 15). The duration between complete liver resection and reperfusion of the autograft was recorded as the anhepatic time. The time of IVC and PV occlusion, including establishment of the temporary IVC and portocaval shunt and reconstruction of the outflow and PV in reimplantation of the autograft, was recorded as the hemodynamic instability time. The secondary endpoints included the overall surgery time and time of each operation stage, actual RLV/SLV, blood loss, blood transfusion, total postoperative stay, and intensive care unit (ICU) stay.

### Statistical Analysis

Continuous data are expressed as the medians and interquartile ranges (Q1–Q3). Categorical data are expressed as numbers and percentages. In this study, we analyzed the learning curve for ELRA using the risk-adjusted cumulative sum (RA-CUSUM) methods.

In this study, the cases were first ordered chronologically (from the first to the last). The learning curve regarding both primary and secondary endpoints were analyzed by time series plots of the raw data and corresponding fitted curves. The interpretation of these plots was based on intuitively visual inspection.

The RA-CUSUM technique plots the difference between the cumulative expected outcome of a categorical variable and the actual observed outcome (16). Univariable logistic regression analysis was conducted to identify factors with a *p*-value < 0.1 that were further entered into a multivariable logistic regression model to assess major postoperative morbidity (defined as a CCI >26). The final model included age, duration between the primary diagnosis and obvious symptoms, remote metastasis, and liver function. Using this model, RA-CUSUM analysis was performed to assess the learning curve for ELRA. The RA-CUSUM plot provides a visual representation of the cumulative incidence of major morbidity regarding the associated risk for a particular case mix. Every operation is plotted from left to right: the line ascends for avoiding postoperative major morbidity, whereas the line descends for encountering postoperative major morbidity. The magnitude by which the line ascends or descends is determined by the difference between the observed and expected proportions of major morbidity. For cases without major morbidity, the line ascends by an amount equal to the estimated probability of major morbidity occurring, and for every case encountering major morbidity, the line descends by an amount equal to 1 minus the estimated probability of major morbidity occurring.

For the sensitivity analysis, once the learning curve based on RA-CUSUM was determined, the entire cohort was divided into different phases that were further tested for the association with all endpoints and further adjusted for risk factors selected by the univariable logistic regression model. A two-tailed *P*-value < 0.05 was considered statistically significant in all analyses. Empower (R) (www.empowerstats.com; X&Y solutions, Inc., Boston MA) and GraphPad Prism 7.0 (GraphPad Prism Software) were used for statistical analyses.

## Results

### Patient Characteristics

**Figure 1:**
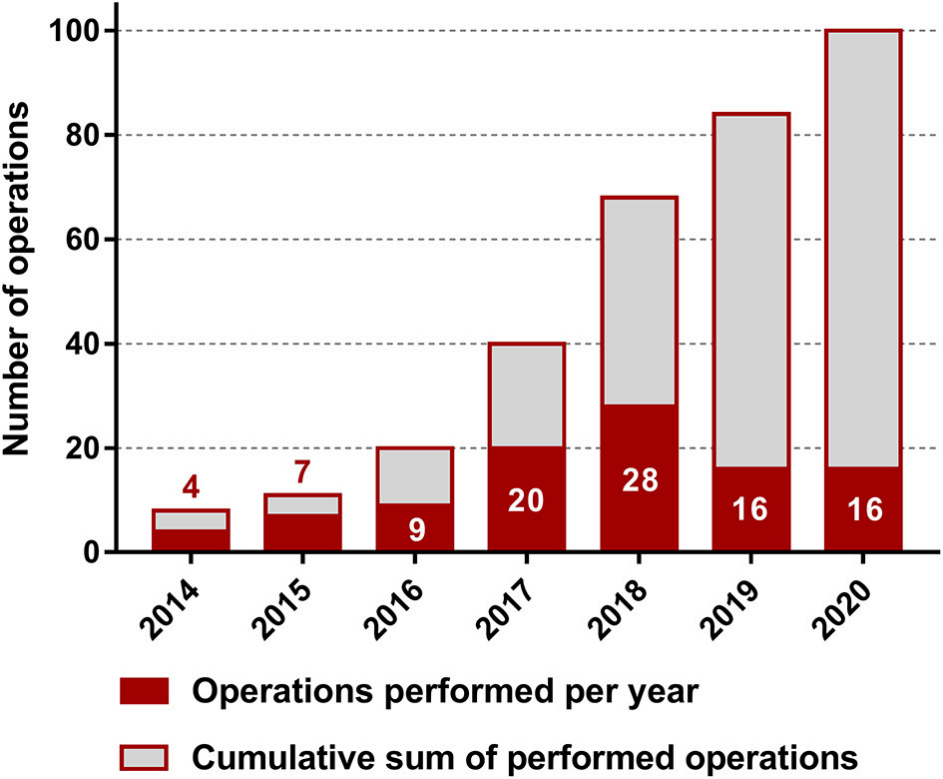
Number of ELRAs completed per year.

The median age of the 102 enrolled patients (38 males and 64 females) was 38 (29–44) years. The median duration between the primary diagnosis and obvious symptoms was 16 (9–38) months. The mean body mass index (BMI) was 21.8 (19.7–23.7). Sixty-six (64.71%) patients received a disciplinary antiparasitic medication before undergoing ELRA, and 18 (17.65%) patients had a history of jaundice. The characteristics of all patients and details of the preoperative assessment are presented in Table 1. The number of cases completed over the years is shown in Figure 1.

**Table 1:** Univariate and multivariate logistic regression analyses of risk factors predicting major complications.

**Variable**	**Value**	**Univariate**	**Multivariate**
**Demographic data**			
Age, year	38 (29–44)	1.04 (1.00, 1.09) **0.0493**	1.03 (0.99, 1.09) 0.1740
BMI	21.8 (19.7–23.7)	0.99 (0.87, 1.12) 0.8839	
Duration between primary diagnosis and obvious symptoms, month	16 (9–38)	1.02 (1.01, 1.04) **0.0018**	1.02 (1.00, 1.03) 0.0236
Sex			
Female	64 (62.75%)	Reference	
Male	38 (37.25%)	0.91 (0.40, 2.08) 0.8235	
Oral medication history			
No	36 (35.29%)	Reference	
Yes	66 (64.71%)	1.38 (0.59, 3.24) 0.4525	
Jaundice history			
No	84 (82.35%)	Reference	
Yes	18 (17.65%)	1.80 (0.65, 5.02) 0.2615	
**Preoperative assessment of lesion anatomy**			
Lesion diameter, cm	15.0 (13.8–16.9)	0.98 (0.84, 1.13) 0.7481	
Neighboring invasion			
No	34 (33.33%)	Reference	
Yes	68 (66.67%)	1.78 (0.74, 4.30) 0.1973	
Remote metastasis			
No	86 (84.31%)	Reference	Reference
Yes	16 (15.69%)	3.28 (1.08, 9.91) **0.0356**	3.42 (1.01, 11.54) 0.0473
Number of lesion			
Single	60 (58.82%)	Reference	
Multiple	42 (41.18%)	0.83 (0.37, 1.88) 0.6613	
Inferior vena cava invasion			
I	8 (7.84%)	Reference	
II	78 (76.47%)	0.99 (0.22, 4.44) 0.9857	
III	16 (15.69%)	1.30 (0.23, 7.38) 0.7699	
Hepatic vein invasion			
I	43 (42.16%)	Reference	
II	59 (57.84%)	1.82 (0.79, 4.17) 0.1577	
Portal vein invasion			
I	7 (6.86%)	Reference	
II	69 (67.65%)	1.42 (0.26, 7.87) 0.6878	
III	26 (25.49%)	2.14 (0.35, 13.12) 0.4097	
**Preoperative assessment of liver quality**			
Estimated remnant liver volume (ERLV), ml	700 (600–840)	1.00 (1.00, 1.00) 0.3793	
Standard liver volume (SLV), ml	1,147 (1,063–1,216)	1.00 (1.00, 1.00) 0.9163	
ERLV/SLV	0.6 (0.5–0.8)	3.47 (0.34, 35.85) 0.2965	
Child-Pugh grade			
Child A	96 (94.12%)	Reference	
Child B	6 (5.88%)	0.80 (0.14, 4.57) 0.7993	
Albumin-Bilirubin grade			
≤-2.60	24 (23.53%)	Reference	Reference
>-2.60	78 (76.47%)	2.94 (1.00, 8.66) **0.0510**	2.23 (0.70, 7.13) 0.1762

*To display the distribution of variables, continuous variable using Median (Q1–Q3), categorical variables using N (%). Results of regression analyses is exhibited as β (95%CI) P-value/OR (95%CI) P-value. Bold values significant p-value that < 0.05*.

### Perioperative Results

Regarding the intraoperative outcomes, 21.57% (22/102) of patients underwent combined resection due to extensive infiltration into adjacent organs and tissue, and the median surgery time was 738 (659–818) min, with a median blood loss of 2,250 (1,600–3,000) ml. The overall incidence of major morbidity was 38.24% (39/102), with a median CCI of 20.92 (0.00–26.22), and the 90-day mortality rate was 10.78% (11/102). The median postoperative stay and ICU stay were 20 (15–31) and 4 (3–5) days, respectively.

Age, duration between the primary diagnosis and obvious symptoms, remote metastasis, and liver function were selected by the univariable logistic regression model and entered into the multivariable prediction model (Table 1). The model had acceptable prediction capability, with an AUC of 0.74 (95% confidence interval [CI] 0.63–0.84) and stable calibration.

### Evaluation of all Endpoints

**Figure 2:**
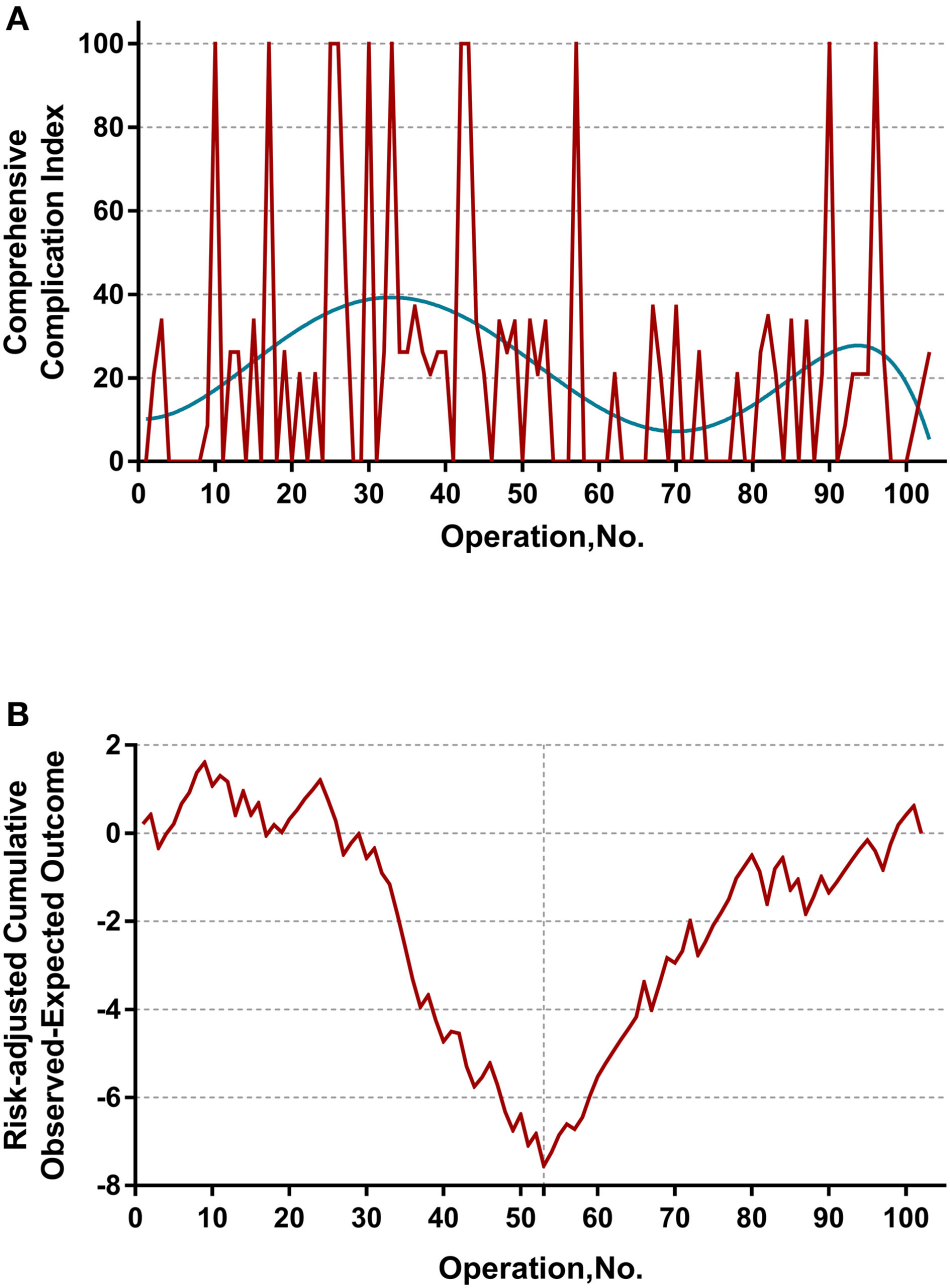
**(A)** Evolution of postoperative complications regarding Comprehensive Complication Index (CCI) was plotted against cases of ELRAs performed. The blue curve represents the curve of best fit for the plots. A visual inspection showed the first peak of fitted curve around case 33 and the second peak around case 95. **(B)** RA-CUSUM analysis of major postoperative morbidity (CCI > 26) for the difference between the cumulative expected outcome and the actual observed outcome. A multivariable logistic regression model for major postoperative morbidity was constructed to calculate the expected outcome. Every operation is plotted from left to right: the line ascends for avoiding postoperative major morbidity, whereas the line descends for encountering postoperative major morbidity. Visual inspection shows a learning curve of 55 procedures.

**Figure 3:**
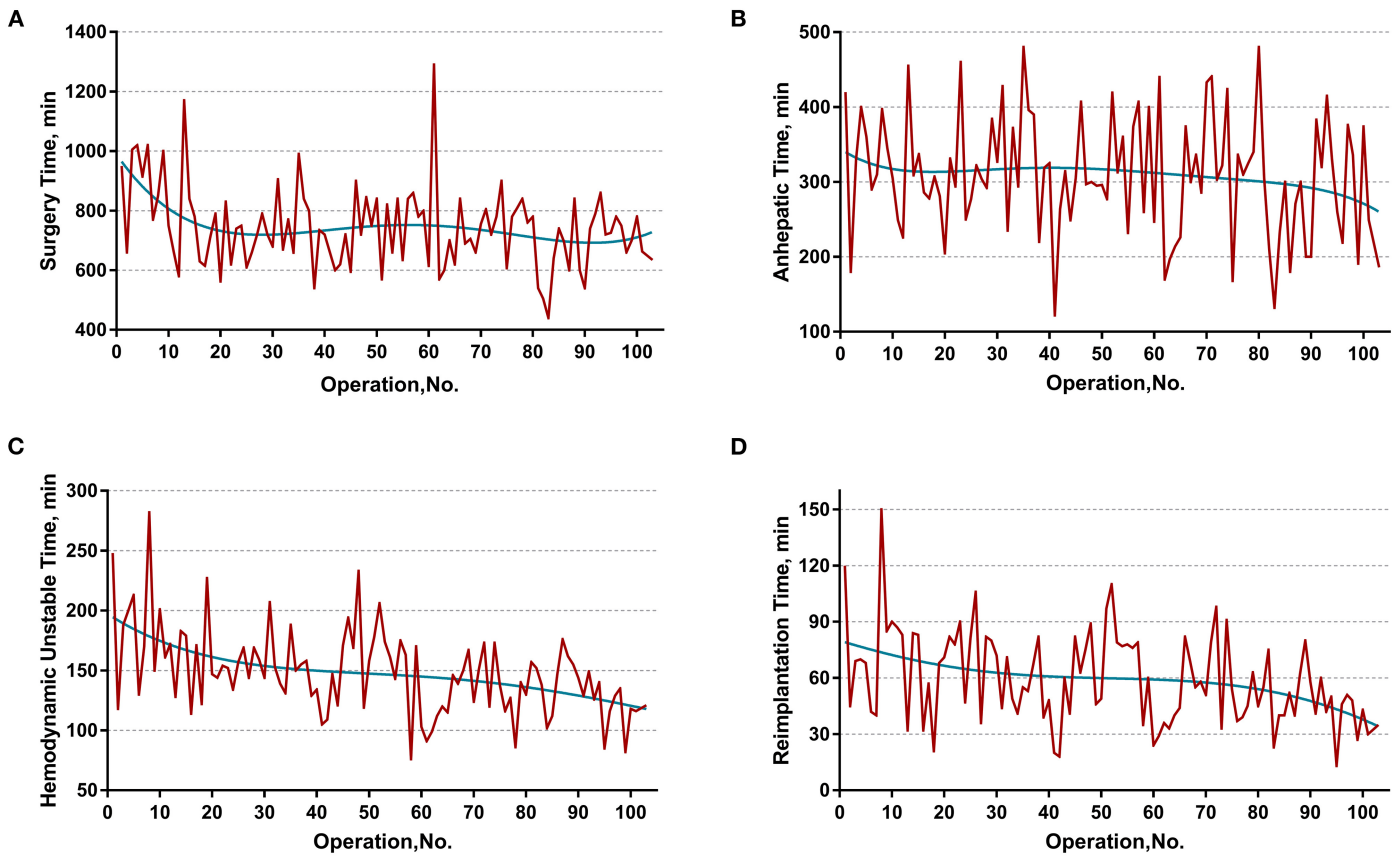
Evolution of overall and staged surgery time in minutes plotted against cases of ELRAs performed. **(A)** Overall surgery time, **(B)** anhepatic time, **(C)** hemodynamic unstable time, **(D)** reimplantation time. The blue curve represents the curve of best fit for the plots. Mild moderating trends were observed in all fitted curves.

**Figure 4:**
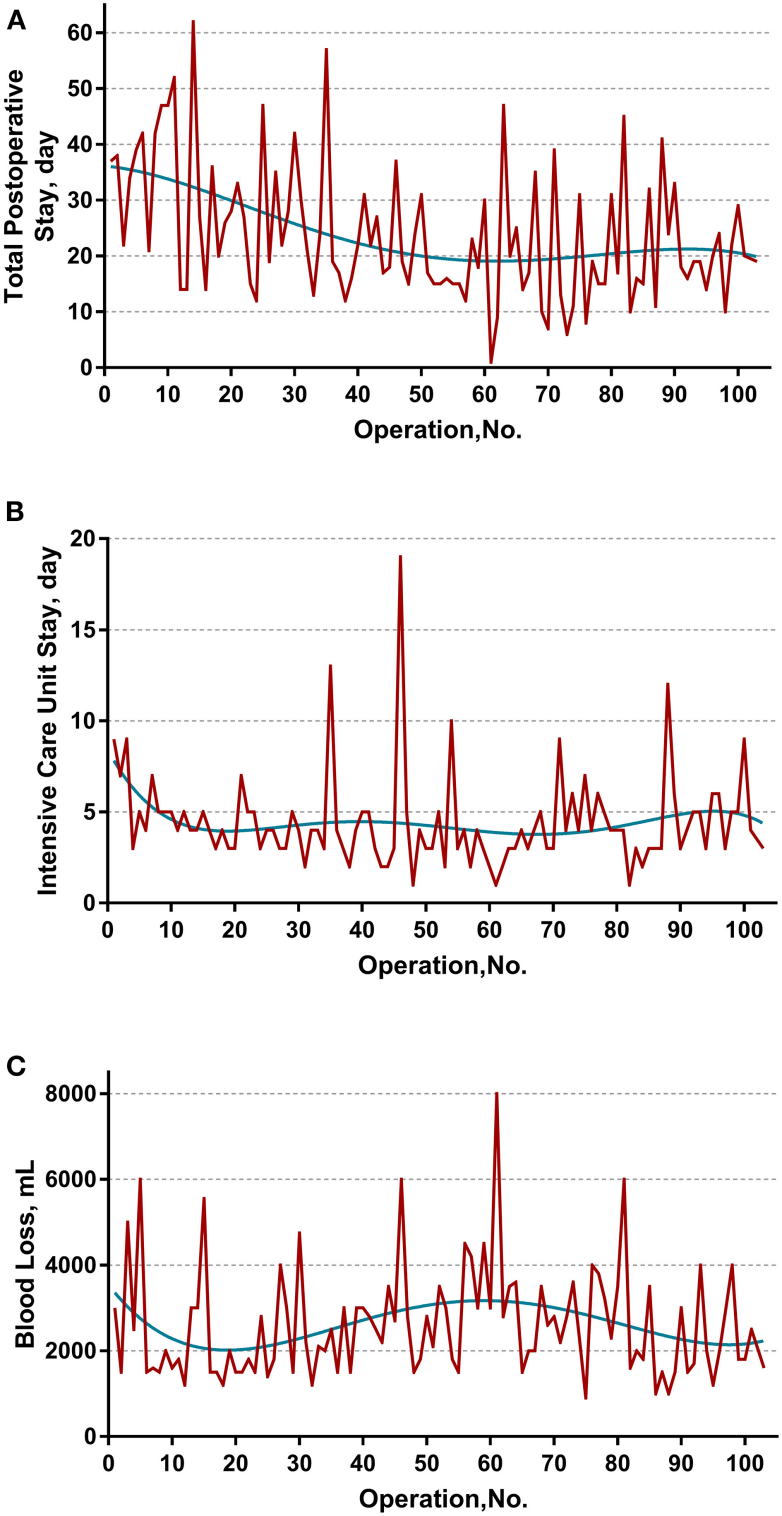
Evolution of other secondary endpoints including **(A)** total postoperative stay, **(B)** intensive unit care stay, and **(C)** blood loss were plotted against cases of ELRAs performed. The blue curve represents the curve of best fit for the plots. No obvious patterns were observed by visual inspection.

The learning curve was first assessed by series plots of the raw data and curve-fitting methods. A visual inspection of the evolution of the CCI (Figure 2A) showed the first peak of fitted curve around case 33 and the second peak around case 95. The first peak of CCI was constituted by the first 8 deceased cases and the second peak was constituted by the mortality of case 90 and 96. Regarding surgery time, the visual inspection of the fitted curves (Figure 3) of the overall surgery time, anhepatic time, hemodynamic unstable time, and reimplantation time showed consistent mild moderating trends. A visual inspection of the plots of the total postoperative stay, ICU stay, and blood loss showed no significant tendency (Figure 4).

### RA-CUSUM Analysis

The learning curve for ELRA was eventually determined by the RA-CUSUM method and is displayed in Figure 2B. A visual inspection of the RA-CUSUM plot showed an increased incidence of major morbidity at the beginning of series that started to decrease after case 53. In other words, case 53 represented the lowest point in the learning curve. For the sensitivity analysis, the entire cohort was divided into Phase I (cases 1–52, *n* = 52) and Phase II (cases 53–102, *n* = 50). The learning phase (Phase II vs. I) showed a significant association with the hemodynamic unstable time [hazard ratio (HR) −30.29, 95% CI −43.32, −17.25, *P* < 0.0001], reimplantation time (HR −13.92, 95% CI −23.17, −4.67, *P* = 0.004), total postoperative stay (HR −6.87, 95% CI −11.33, −2.41, *P* = 0.0033), and postoperative major morbidity (HR 0.25, 95% CI 0.09, 0.68, *p* = 0.007) when adjusted for age, disease course, liver function, and remote metastasis. No significant association with other endpoints was observed (Table 2).

**Table 2:** Association of learning phases with intraoperative and postoperative outcomes.

**Variable**	**Value**			**Association with learning Phases (Phase II vs. I)**					
	**Overall**	**Phase I**	**Phase II**	**Non-adjusted**			**Adjusted**		
	**(*n* = 102)**	**(*n* = 52)**	**(*n* = 50 cases)**		
**Intraoperative outcomes**									
Combined resection				0.66	(0.25, 1.71)	0.3919	0.56	(0.18, 1.76)	0.3205
No	80 (78.43%)	39 (75.00%)	41 (82.00%)						
Yes	22 (21.57%)	13 (25.00%)	9 (18.00%)						
Actual RLV/SLV	0.6 (0.5–0.7)	0.6 (0.5–0.7)	0.7 (0.5–0.8)	0.08	(0.01, 0.15)	**0.0201**	0.07	(−0.00, 0.14)	0.0649
Surgery time, min	738 (659–818)	745 (660–840)	723 (636–787)	−39.09	(−91.93, 13.76)	0.1502	−31.94	(−81.28, 17.40)	0.2077
Anhepatic time, min	306 (253–369)	306 (280–363)	304 (227–370)	−17.82	(−48.50, 12.87)	0.2578	−15.27	(−46.03, 15.49)	0.333
Hemodynamic unstable time, min	147 (125–169)	157 (137–180)	137 (116–152)	−29.69	(−42.15, −17.24)	** <0.0001**	−30.29	(−43.32, −17.25)	** <0.0001**
Reimplantation time, min	55 (41–78)	69 (46–82)	49 (39–63)	−14.35	(−23.37, −5.32)	**0.0024**	−13.92	(−23.17, −4.67)	**0.004**
Blood loss, ml	2,250 (1,600–3,000)	2,100 (1,500–3,000)	2,550 (1,800–3,500)	253.00	(−240.58, 746.58)	0.3175	403.96	(−83.44, 891.37)	0.1076
Autologous blood transfusion, ml	775 (0–1,325)	550 (0–1075)	825 (500–1,500)	292.36	(−82.98, 667.70)	0.13	366.00	(−31.16, 763.15)	0.074
Red blood cell suspension transfusion, U	6 (4–10)	7 (4–10)	6 (4–9)	30.19	(−31.09, 91.47)	0.3366	42.51	(−22.40, 107.43)	0.2024
Frozen plasma transfusion, ml	600 (400–1,192)	600 (225–1,600)	640 (400–950)	−326.61	(−714.87, 61.66)	0.1023	−213.96	(−614.71, 186.79)	0.298
**Postoperative outcomes**									
Total postoperative stay	20 (15–31)	25 (18–36)	18 (14–24)	−7.99	(−12.40, −3.58)	**0.0006**	−6.87	(−11.33, −2.41)	**0.0033**
Intensive care unit stay	4 (3–5)	4 (3–5)	4 (3–5)	−0.38	(−1.36, 0.61)	0.4588	−0.28	(−1.31, 0.75)	0.5971
Major morbidity				0.37	(0.16, 0.84)	**0.0179**	0.25	(0.09, 0.68)	**0.007**
CCI ≤26	63 (61.76%)	26 (50.00%)	37 (74.00%)						
CCI >26	39 (38.24%)	26 (50.00%)	13 (26.00%)						

*RLV, remnant liver volume; SLV, standard liver volume; CCI, comprehensive complication index. Association between learning phases and each surgical outcome was tested by logistic regression, respectively, and consequently adjusted for age, course of disease, liver function, remote metastasis. To display the Distribution of variables, continuous variable using Median (Q1–Q3), categorical variables using N (%). Results of regression analyses was exhibited as β (95% confidence interval) P-value/OR (95% confidence interval) P-value. Bold values significant p-value that <0.05*.

## Discussion

To our knowledge, this study is the first analysis of a learning curve in a large series of patients with end-stage HAE treated by ELRA. With RA-CUSUM learning curve analysis, a learning curve of 53 operations for major postoperative morbidity was demonstrated. Based on a median surgery time of 738 (659–818) min, blood loss of 2,250 (1,600–3,000) ml, major postoperative morbidity rate of 38.24% (*n* = 39), and 90-day mortality rate of 10.78%, ELRA is considered a safe procedure within a highly experienced transplantation surgical team.

Alveolar echinococcosis is a major public health burden in relatively endemic areas (16). China alone accounts for 90% of the global burden of AE, with over 16,000 cases diagnosed every year (17, 18). Due to its insidious onset and slow progression, HAE usually results in a delayed diagnosis and a >90% mortality rate in untreated patients (17, 18). Unfortunately, only 20–40% of HAE patients are eligible for radical hepatectomy (19, 20) and lack the opportunity to receive liver allotransplantation due to a shortage of graft donors, high treatment expenses, and a risk of recurrence associated with mandatory immunosuppressive therapy (21–23). Given that there is a relatively long time between primary infection and the presentation of HAE symptoms enabling adequate compensatory function of the disease-free lobe of the liver, surgeons tend to perform complex and risky procedures, such as ELRA, to accomplish radical treatment under the condition that an adequate RLV and the feasibility of reconstruction are certain.

Developed by Rudolf Pichlmayr and his team, ELRA was primarily utilized in the treatment of “unresectable” hepatobiliary malignant tumors and has been reported to be a promising alternative to liver allotransplantation in treating end-stage HAE (8, 24, 25), a chronic tumor-like infectious disease that is highly sensitive to immunosuppressive therapy (26, 27). Regarding the treatment of end-stage HAE, the difficulty of ELRA is associated mainly with the following: (1) the enlarged scale of combined resection caused by extensive lesion invasion; (2) the identification and preservation of abnormal vessels under a bloodless circumstance during bench resection and simultaneous radical resection of the liver parenchyma; (3) highly individualized plasty and reconstruction of impaired vessels; and (4) maintenance of hemodynamics and homeostasis during the operation. Hampered by these technical obstacles, the feasibility, safety and actual benefit of ELRA were inevitably disputed (28). Our center managed these difficulties by a meticulous preoperative assessment and planning and abundant experience in transplantation and vascular surgery and achieved an acceptable outcome.

The main finding of the present study is the determination of a learning curve based on the performance of postoperative morbidity. On visual inspection of the plots demonstrated in Figure 2A, most mortalities (CCI = 100) were clustered in first 50 cases. However, the learning curve of ELRA cannot be determined by simple inspection of the evolution of CCI raw data because the underlying risk originating from the inherent features of each case is not considered. Thus, an RA-CUSUM chart adjusted for age, disease course, liver function and remote metastasis was constructed. In the presented cohort, case 53 represented the point at which the increasing tendency of postoperative complication incidence was reversed. This point could be considered the point at which competence in ELRA was achieved.

Further sensitivity analysis, by distinguishing two learning phases from the entire cohort, displayed a consistent impact of learning phases on the hemodynamic unstable time, reimplantation time, total postoperative stay, and major morbidity. Intriguingly, the learning phase had no significant impact on the overall surgery time. This might be the result of a drastically varied anhepatic time. The anhepatic time in conventional liver allotransplantation represents the duration between complete resection of the recipient liver and revascularization of the donor liver and the duration of unstable hemodynamics with an occluded IVC and congested gastrointestinal system. However, the anhepatic time in ELRA also includes the duration of *ex vivo* liver resection, while the hemodynamics are maintained by the temporary IVC and portocaval shunt. This period is mainly determined by the time consumed for bench resection and tailoring vessels. These highly individualized procedures are mostly influenced by the heterogeneous extension of HAE lesions instead of the expertise of the surgical team. Thus, the learning phase had no significant impact on the anhepatic time or overall surgery time. On the other hand, the unstable hemodynamic time and reimplantation time are basically determined by the proficiency of vascular reconstruction, leading to a significant improvement in different learning phases.

The techniques and materials chosen for vascular reconstruction may also significantly affect the surgery time. We ultimately select the materials of IVC reconstruction intraoperatively based on the preoperative evaluation and intraoperative findings. Due to concerns about the potential risk of graft failure caused by infection and histocompatibility, autologous materials were preferable. The cryopreserved homologous vascular grafts are also recommended previously (29). Autologous and homologous materials take advantage of histocompatibility and flexibility of tailoring while possibly increasing the operation time. In some extreme cases of absolute IVC stenosis, compensatory collateral circulation was fully established during the lengthy onset of HAE. Thus, the outflow was directly anastomosed to the vena cava foramen, leaving the IVC unconstructed. Several reports emphasized that the presence or absence of venous collateral circulation is an essential indicator of the need for IVC reconstruction (30, 31), which highly agrees with our opinion.

By interpreting the results of the RA-CUSUM analysis, we recognized that when referring to a learning curve, a conclusion cannot be based on a single outcome, such as the incidence of major morbidity. Additional endpoints, such as postoperative stay and blood loss, should also be assessed, although there is no clear definition of which variables constitute a learning curve. The RA-CUSUM method does not allow the learning curve of continuous variables to be calculated. Therefore, series plots of the raw data and curves-fitting methods were used for evaluating other endpoints. On the other hand, the widely used conventional CUSUM analysis, which calculates the sequential difference between the raw data and the mean value was not used in this study. As discussed by Woodall et al. (32), the CUSUM learning curve approach based on accumulating the differences between the performance metric values and their average value can be misleading and is often misinterpreted. Thus, we analyze the values of all endpoints directly using curve-fitting methods. Unfortunately, no clear conclusion can be drawn on secondary endpoints in either the plots or consequent sensitivity analysis.

Contrary to the current tendency of minimally invasive techniques, ELRA represents a pinnacle of hepatobiliary open surgery, which may be regarded as a “resurgence” of conventional open surgery. The difficulties of ELRA mentioned above must be overcome to safely perform ELRA. First, unlike the general process of procuring the liver in allogeneic LT, serious infiltration to adjacent organs and tissues is frequently encountered in ELRA. This requires that the surgeon be a fortified hepatobiliary surgeon with additional expertise in thoracic and retroperitoneal operations. Second, in contrast to a normal donor liver, a diseased liver treated by ELRA has abnormal intrahepatic vessels severely infiltrated by HAE and frequently complicated by vascular malformation and cholangiectasis. This predicament further increases the difficulties of dissecting the liver parenchyma during *ex vivo* resection, and its management requires abundant experience from surgeons in living-donor and split LTs. Third, for cases with elevated serum bilirubin led by a biliary obstruction, appropriate preoperative management is warranted to obtain the operation chance. The intraoperative frozen section examination is also necessary to further affirm the quality of the residual liver. The additional pathological examination on remanent liver parenchyma may be helpful for early warning of postoperative liver function related complications (33), while some reports underrate the value of such endeavor (34). Finally, the highly individualized vessel reconstruction technique remains the bottleneck of ELRA. It is reasonable to say that based on the experience of our center, there are almost no identical reconstruction strategies. Even with the aid of an advanced imaging evaluation, it is still impossible to fully predict variation in the conduits and damage that may be encountered during the operation, which cause great difficulties in the repair and reconstruction strategy. To address such problems, we developed a classification based on vascular infiltration (9) that focuses on improving the comprehension of lesion anatomy and facilitating surgical planning and obtained acceptable effectiveness. In summary, unlike other surgeries that have been or are being widely promoted, ELRA might be utilized in large transplantation centers by a multidisciplinary team (MDT) that includes a hepatobiliary surgeon, vascular surgeon, radiologist, and anesthetist with abundant experience in multiple LT modalities (living donor, split, auxiliary, etc.). However, for centers preparing for ELRA, the patient volume may be insufficient for approaching the demand of the learning curve over a certain period of time due to the limited indications for this surgery.

This study had some limitations. The limited number of patients may have abated the power of the regression analysis for identifying risk factors with the RA-CUSUM method. The retrospective design may have introduced selection bias. Consequently, the results of our study apply only to this specific cohort. The strengths of this study were that it was conducted on the largest cohort of patients with end-stage HAE to date, and ELRA was conducted by the same surgical team. Moreover, this was the first study on the learning curve of ELRA representing the course of managing this extremely complex surgery by a single experienced transplantation team and may provide a reference for other complex liver surgeries. The promising results of ELRA propagate further prospective and randomized trials on the actual benefits of ELRA.

## Conclusions

This study demonstrated the feasibility and safety of ELRA in treating end-stage HAE. When performed by a multidisciplinary transplantation surgical team led by a senior hepatobiliary surgeon with abundant experience in thoracic and retroperitoneal operations and living-donor and split LTs, the inherent benefits of ELRA were not compromised in treating end-stage HAE. A learning curve of 53 cases is achievable when these conditions are met.

## Data Availability Statement

The raw data supporting the conclusions of this article will be made available by the authors, without undue reservation.

## Author Contributions

YQ, BH, and WW participated in research design. YQ participated in the writing of the paper. YQ, XY, TW, and SS participated in the performance of the research. YQ and YY participated in data analysis. All authors contributed to the article and approved the submitted version.

## Funding

This research was supported by the Science and Technology Program of Sichuan Science and Technology Department (Nos. 2019YFS0029 and 2019YFS0529), the National Natural Science Foundation of China (Nos. 81770566 and 82000599), and the New Medical Technology Foundation of West China Hospital of Sichuan University (No. XJS2016004). WW is the guarantor. The funding body financed the costs of the study and contributed to the design of the study, interpretation of data, and revision of the manuscript.

## Conflict of Interest

The authors declare that the research was conducted in the absence of any commercial or financial relationships that could be construed as a potential conflict of interest.

## Publisher's Note

All claims expressed in this article are solely those of the authors and do not necessarily represent those of their affiliated organizations, or those of the publisher, the editors and the reviewers. Any product that may be evaluated in this article, or claim that may be made by its manufacturer, is not guaranteed or endorsed by the publisher.

## Abbreviations

ELRA: *ex vivo* liver resection and autotransplantation
LT: liver transplantation
IVC: inferior vena cava
PV: portal vein
HV: hepatic vein
HAE: hepatic alveolar echinococcosis
*Em*: *Echinococcus multilocularis*
AE: alveolar echinococcosis
RLV: remnant liver volume
SLV: standard liver volume
CCI: comprehensive complication index
RA-CUSUM: risk-adjusted cumulative sum.
